# A methodological approach for using high-level Petri Nets to model the immune system response

**DOI:** 10.1186/s12859-016-1361-6

**Published:** 2016-12-22

**Authors:** Marzio Pennisi, Salvatore Cavalieri, Santo Motta, Francesco Pappalardo

**Affiliations:** 1 Department of Mathematics and Computer Science, University of Catania, Catania, Italy; 2 Department of Electrical Electronic and Computer Engineering (DIEEI), University of Catania, Catania, Italy; 3 Department of Drug Sciences, University of Catania, Catania, Italy

**Keywords:** Computational modeling, Systems biology, Petri Nets, Immunity, Immune system, Design methodology, Humoral response

## Abstract

**Background:**

Mathematical and computational models showed to be a very important support tool for the comprehension of the immune system response against pathogens. Models and simulations allowed to study the immune system behavior, to test biological hypotheses about diseases and infection dynamics, and to improve and optimize novel and existing drugs and vaccines.

Continuous models, mainly based on differential equations, usually allow to qualitatively study the system but lack in description; conversely discrete models, such as agent based models and cellular automata, permit to describe in detail entities properties at the cost of losing most qualitative analyses. Petri Nets (PN) are a graphical modeling tool developed to model concurrency and synchronization in distributed systems. Their use has become increasingly marked also thanks to the introduction in the years of many features and extensions which lead to the born of “high level” PN.

**Results:**

We propose a novel methodological approach that is based on high level PN, and in particular on Colored Petri Nets (CPN), that can be used to model the immune system response at the cellular scale. To demonstrate the potentiality of the approach we provide a simple model of the humoral immune system response that is able of reproducing some of the most complex well-known features of the adaptive response like memory and specificity features.

**Conclusions:**

The methodology we present has advantages of both the two classical approaches based on continuous and discrete models, since it allows to gain good level of granularity in the description of cells behavior without losing the possibility of having a qualitative analysis. Furthermore, the presented methodology based on CPN allows the adoption of the same graphical modeling technique well known to life scientists that use PN for the modeling of signaling pathways. Finally, such an approach may open the floodgates to the realization of multi scale models that integrate both signaling pathways (intra cellular) models and cellular (population) models built upon the same technique and software.

## Background

The immune system (IS) is a highly distributed system composed by cells and molecules whose scope is to protect living organisms against diseases. The immune system has various defense mechanisms whose complexity is usually correlated to the host organism. In mammals, among many defense mechanisms such as innate immune response arm, the adaptive IS response, driven by lymphocytes, represents a fundamental component. The goal is of the adaptive IS response is to give the best reaction against most dangerous viruses and bacteria.

Specificity, memory and discrimination between self and non-self represent the most important peculiarities of the adaptive IS response. In particular, the specificity feature is the capability, shared only by lymphocytes and antibodies, to recognize one specific epitope (also known as antigenic determinant) of foreign pathogens [1]. Specific immune system lymphocytes can then recognize billions of different antigens, but for each determinant, a specific lymphocyte will be stimulated. There are as many stimulated lymphocytes as determinants forming the antigen.

Indeed, the memory feature represents the capability of the immune system to remember previously encountered antigens. When an antigen appears for the first time, the immune system produces a first (primary) response against it. This stimulates the production of long-lived memory lymphocytes that are specific for each of the antigen epitopes. When the same antigen comes again into contact with the immune system, the memory lymphocytes are stimulated in order to produce a faster and more effective immune system response against it (secondary response).

Building a model (mathematical, physical or computational) of the immune system may lead to multiple benefits, since researchers can use immune system simulation in vaccine research, or to test biological hypotheses about diseases and infection dynamics.

Many mathematical and computational techniques have been applied to model the immune system response; most of them bear on two main approaches: the top-down approach and the bottom-up approach. If the former mainly focuses on the behavior of a system as a whole (i.e., mean behavior of involved cells), the latter analyzes the system dynamics at a lower level (i.e., the behavior of every cell is kept into account) and the emergence of global properties is obtained as the sum of local behaviors. Models based on the top-down approach usually make use of differential equations like Ordinary Differential Equations (ODE) [2–8]. These models are all population-based, and the spatiality and topology which both depend on individual interactions are, in general, ignored. For very simple models it is possible to use some powerful mathematical techniques to extract some important system properties like analytical solutions, steady states and asymptotic behavior. However, as the biological description introduced into the model grows, they become very complex and, in some cases, almost intractable. Indeed, the application of Partial Differential Equations (PDE), stochastic differential equations (SDE) and delayed differential equations (DDE) can be introduced to obtain some spatial description, stochastic effects and temporal delays. However, this usually entitles instability problems, a higher computational effort and numerical simulations only.

Cellular automata and agent based models represent the most famous bottom-up approaches. Since entities are followed individually, they allow a much more rich description of the biological background, and individual cells properties like cell receptors, cell internal states and individual life-times and behaviors are not a problem. Furthermore, spatial description, stochastic effects and delays are intrinsic characteristics of these kind of models. However, such techniques lack of tools and mathematical techniques for analytical studies and they usually require huge computational resources for large scale simulations.

Petri Nets (PNs) represent a graphical and mathematical modeling technique for the description of distributed systems presented in 1962 by Carl Adam Petri [8, 9]. During these last decades, Petri Net modeling formalism has been extended in order to manage, among others, stochastic effects, continuous quantities and scheduled events. Among PN extensions there is the Colored Petri Nets (CPN) formalism [10].

Petri Nets and extensions have been successfully used in bioinformatics for the modeling of intra-cellular biochemical and metabolic pathways [11–18]. Park et al. showed how it is possible to apply a particular extension of PN (the Fuzzy continuous PN) for the modeling of the Immune system response [19]. The approach had several limitations; among them, it did not allow to discern between cells featuring different internal states, receptors, or a different position inside the simulation space. Consequently, such an approach did not bring any real additional advantage over up-down traditional techniques such as ODE systems, and neither in respect to ABMs.

Here we propose a novel methodological approach that uses “high level” Petri Nets, and in particular Colored Petri Nets to model cell specific properties such as cell internal states and receptors. To this end we present a proof-of-concept model of the humoral part of the IS capable of reproducing the most important features of the adaptive response, and in particular the memory and specificity features. The approach holds somewhere in the middle of top-down and bottom-up approaches, since it allows to describe cell specific features and behaviors, but with the possibility of using many standard PN analysis tools that permit to study system properties and features from a qualitative point of view. All of this using a convenient graphical language that is already known to life scientists that, as already stated, are actually able to use PN for the modeling of signaling pathways.

The paper starts with an introduction to Petri nets and the colored extension used in this paper. Details of the modeling approach are provided in Methods, followed by results and discussion where we present the application of the approach to a real scenario, ending with our conclusions and final remarks.

## Methods

### Introduction to classical “low-level” Petri Nets

PNs is a set of particular graphical symbols called: places, transitions, arcs and tokens. The places are generally drawn as ellipses or circles and represent states and/or conditions of the system modeled; each place may be featured by a name (many times inside the ellipses), which has not a formal meaning but increases understanding of a Petri Net. Boxes or rectangular bars represent transitions which model events that may occur in the system. Places usually contain a discrete number of elements called tokens, drawn by black dots. Arcs may connect places to transitions and vice-versa, but they cannot connect transitions to transitions or places to places. An arc connects a single (input) place to a single transition; furthermore, an arc may connect a transition to a single (output) place. A transition may have several input and output places. The tokens in the input places are fundamental for the firing (i.e., the enabling) of the transition; the enabling of a transition models the occurrence of a specific event. Firing of a transition may require the presence of a different number of tokens for each input place. Generally, the required number of tokens for each input place is given by the multiplicity of each arc connecting input places with the transition. When a transition fires (i.e., there are sufficient tokens in all of its input places, according to the multiplicities of the arcs), it consumes the required number of tokens from the input places, and creates tokens in its output places. The number of tokens put in each output place depends again by the multiplicity associated to each arc. A firing rule is atomic, in the sense that it represents a single non-interruptible action. Unless an execution policy is defined, when multiple transitions can be enabled at the same time anyone of them may fire (nondeterministic execution).

### Introduction to “high-level” Colored Petri Nets

A Colored Petri Net (CPN) extend the definition of Petri Nets as it is a set of places, transitions, arcs and color sets. Each place has an associated **type** determining the kind of data that the place may contain (by convention the type name is written in italics, next to the place). Like PNs, during the execution of a CPN (i.e., the firing of the transitions) each place will contain a varying number of tokens. Each of these tokens carries a data value that belongs to the type associated with the place. For historical reasons, token values are referred as token **colors** and data types are referred as **color sets**. This is a metaphoric picture where the tokens of “high-level” CPN are considered to be distinguishable from each other and hence “colored” – in contrast to ordinary “low-level” Petri Nets which have “black” indistinguishable tokens. The types of a CPN can be arbitrarily complex e.g., a record where one field is a real, another a text string and a third a list of integers. Hence, it is much more adequate to imagine a continuum of colors (like in physics) instead of a few discrete color values (like red, green and blue).

In CPNs, there are different types of expressions. An expression is mainly made up by variables. It is not only associated with a particular color set but also written in terms of a predefined syntax. In the following, we denote by EXP a set of expressions that comply with a predefined syntax. An expression may be associated to a transition; in this case, it is called **guard expression**. An arc may have an expression associated, too; it is called **arc expressions**.

On the basis of what said until now, the formal definition of colored Petri Nets will be given in the following. It uses the concept of **multiset**, which can be defined as a set in which there can be several occurrences for the same element. The number of occurrences of an element is called coefficient or multiplicity. For example, an infinite number of multisets exist which contain elements a and b, varying only by multiplicity, such as: {a, b}, {a, a, b}, {a, a, a, b, b, b}.

In the following, given a generic set *E*, the collection of all the multisets over *E* is denoted by *E*_*MS*_.

**Definition**: A Colored Petri Net is a tuple N = (S, P, T, A, C, G, F, i) where:

- S is a finite non-empty set of types, called **color sets**.
- P is a finite non-empty set of **places**.
- T is a finite non-empty set of **transitions**.
- A is a finite set of direct **arcs**
- C is a **color** function that assigns to each place p∈P a color set C(p)∈S. C: P → S
- G is the set of g functions that assigns to each transition t∈T a guard expression of the boolean type. g: T → EXP
- F is the set of f function that assigns to each arc a∈A an arc expression of a multiset type C(p)_MS_, where p is the place connected to the arc a. f: A → EXP
- i is an initialization function that assigns to each place p∈P an expression of a multiset type C(p)_MS_. i: P → EXP

According to the tokens distribution in the set p∈P, the CPN may assume different states; each state is called marking and is given by a particular token distribution in each of the places p∈P. The initial marking is produced by the application of the initialization function i. In the following the current token distribution in a particular place will be indicated by **m**(p), which is a function of a multiset type C(p)_MS_.

Given a particular a transition t ∈ T, let us consider the set of variables present in the guard of t and in the arc expressions of arcs connected to t; this set will be denoted Var(t). Before the guard and arc expressions relevant to the transition t ∈ T may be evaluated, the variables must get assigned values; this process is called binding. A binding **b** of a transition t ∈ T is a function that maps each variable v ∈ Var(t) onto a value b(v) that is of the same type as the variable. The set of all bindings for a transition t is denoted B(t). A binding element is a pair (t,b), with t ∈ T and b ∈ B(t).

The behavior of the CPN is based on a firing rule consisting of a precondition and the effect of the occurrence (firing) of a single transition. Whether or not a transition can fire depends on the marking of its input places and the arc expression on the input arcs. A transition is enabled and is allowed to fire, if all the input places are sufficiently marked; this occurs when the binding of the variables that appear in the arc expressions of the arcs from inputs places to transition, evaluates to a multiset of token colors (i.e., of multiset type C(p)_MS_) that is present on the corresponding input place. In this case, the guard of the transition should evaluate to true for the giving binding. If a transition has no input places, it is always enabled. In a more formal way, it is possible to say that the guard of the transition g(t) ∈ G evaluates to true if m(p) ≥ f(p,t), ∀p ∈ •t, where f ∈ F and •t is the set of input places for transition t. Due to the definition of set F, f(p,t) denotes the value (of multiset type C(p)_MS_) of the arc expression relevant to the arc going from p to t, for the giving binding b.

If the guard of the transition evaluates to true (with a given binding), the firing of a transition occurs. In this case, a multiset of colored tokens are taken from each input place and added to output places in accordance with the values given by the arc expressions relevant to the arcs connecting the transition to output places. This means that the firing process lead to a change in the current marking of the CPN. In particular, the current marking m becomes the new marking m’, defined by m’(p) = m(p) + f’(t,p)-f(p,t), where f’(t,p) ∈ F is evaluated for ∀p ∈ t• (i.e., the set of output places for transition t) and denotes the values of the arc expressions relevant to the arcs going from p to t, for the giving binding b.

A binding element may occur concurrently to other binding elements – iff there are so many tokens that each binding element can get its “own share”.

### The Colored Generalized Stochastic Petri Net framework extension

Different CPN modeling paradigms have been defined during years: colored qualitative Petri Nets (*QPN*_*C*_), colored stochastic Petri Nets (*SPN*_*C*_), and colored continuous Petri Nets (*CPN*_*C*_) [20, 21]. *QPN*_*C*_ does not uses tokens but it is only used for qualitative analysis of systems; *SPN*_*C*_ takes into account discrete tokens and stochastic transitions, and *CPN*_*C*_ uses continuous quantities rather than discrete tokens and continuous transition rates instead of discrete transitions. *QPN*_*C*_ is an abstraction of *SPN*_*C*_ and *CPN*_*C*_, while *SPN*_*C*_ can reproduce by approximation *CPN*_*C*_ and vice-versa. From our point of view, the most interesting approach is represented by *SPN*_*C*_. In brief, this colored PN extension uses a discrete number of tokens on its places like in classical PNs (cells, for example, are better represented by discrete quantities rather than continuous quantities like in ODE systems). Moreover, stochastic events and transitions that are fundamental for simulating, for example, different individuals, are flawlessly reproduced through the use of stochastic transitions which fire after a probabilistic delay determined by a random variable defined according an exponential probability distribution.

For our purposes it is useful to consider a further extension of the *SPN*_*C*_ framework, namely **Colored Generalized Stochastic Petri Nets**, by considering, besides of stochastic transitions, also some kinds of deterministic transitions that we can identify as immediate, delayed, and scheduled transitions.

All transitions become enabled if all the preplaces are sufficiently marked. When a stochastic transition (represented in our model by a white box) is enabled, a given time must be wait before the firing occurs. This waiting time that determines the firing delay of the transition is given by a random variable *X*_*t*_ that is distributed exponentially with the following probability density function:

$$ {f}_{Xt}\left(\tau \right)={\uplambda}_t(m)\cdot {\mathrm{e}}^{\left(-{\uplambda}_t(m)\cdot \tau \right)},\kern1em \tau \ge 0. $$

Where λ_t_ can be defined as an arbitrary mathematical function which depends on the marking *m* of the preplaces at time *t* (i.e. mass action law that depends on the number of tokens in the preplaces).

It should be noted that, even if there is a stochastic time delay before the firing of the transition, the firing itself does not consume any time.

Deterministic (delayed) transitions (represented by black boxes) have a deterministic firing delay specified by an integer number. The delay count starts just after the transition becomes enabled. However, it must be said that during this waiting time it can happen that the transition loses its enabled state (pre-emptive firing rule).

Immediate transitions (represented by black rectangles as in the standard PN notation) can be seen as a special case of Deterministic (delayed) transitions with a delay time set to 0. In case of conflict between an immediate transition and any other kind of transition, the former will get firing priority.

Also Scheduled transitions (represented in by gray boxes) can be seen as a special case of deterministic transitions. The firing is deterministic and it occurs at a previously defined absolute time of the simulation, obviously only if the transition is enabled at that time. Interested readers can find further details about *SPN*_*C*_ [22, 23].

### Advantages of using Colored Petri Nets

Petri Nets represent a graphical modeling tool that allows to describe in a simple and clear, but yet formally correct and powerful manner, any kind of process. The biggest problem of classical low-level Petri Nets is given by the fact that they usually not scale. As the (biological) process we want to describe grows, low-level Petri Nets tend to grow quickly, and the designing, drawing, understanding and managing of the net becomes even more and more difficult, thus increasing the developing time and the risk of introducing modeling errors.

The introduction of “colors” allows to discern among different tokens and to follow the individually, in a similar way as it is done by CA and ABM approaches. This represents the most important feature of Colored Petri Nets, and their greatest advantage in respect to low-level PNs, but also in respect to all the differential equation based models.

Just to give to the reader an idea of the practical advantage of CPNs, the proof-of-concept model that we developed according to the approach described in the next section required only 9 places, 27 transitions and 63 arcs. If a standard (non colored) PN approach would have been used, it would have required approximately 4000 different places, 28,000 transitions and 84,000 arcs. This means that the development of the model itself would have been almost unfeasible. A similar scenario holds, for example, if a standard ODE approach is used (approx. 4000 equations to describe all the populations and approx. 84,000 nonlinear interaction terms within equations).

The biggest advantage of CPNs (and PNs in general) in respect to classical bottom up approaches is given by the availability of a large number of formal analysis methods by which the important properties of the net can be extrapolated, proved and analyzed. Liveness, Boundedness, Place invariants, Circuits, T-Invariants and so on [8], allow to study the net properties from a qualitative point of view, making the PN approach more similar to a differential equation based approach in this sense. Furthermore, over classical simulation, it is possible to have interactive simulations with results drawn directly and in real time on the CPN diagram, and it is possible to act during these simulations by, for example, introducing and modifying new and existing tokens. This kind of interactive simulation allows to debug the model behavior while it is developed, just like in a complete programming language environment.

Indeed, novel simulation techniques that should speedup and potentially allow large scale simulations as, for example, simulations that use the “symbolic marking” of the net or “fluidification” techniques have now been presented [24, 25] and their implementation in present PN software is in due course.

Finally, CPNs (and PNs in general) have a graphical representation. This graphical representation is very simple, intuitive, and very appealing, even for people who are not very familiar with the formal details of CPN. With only three kinds of graphical items (circles, boxes, and arrows) it is possible to mostly describe any kind of process and to obtain, for free, a very clear and qualitative conceptual model of process itself. Arc and transitions guards can include very simple first-order logic rules, as well as more complex functions that can be defined using look-up tables. Thus, nor a strong knowledge of mathematics (as for differential equation based models), neither a good knowledge of any programming language (as for CA for ABM models) is required, and this may represent a consistent leap ahead in respect to classical approaches, since it could allow to life-scientists to directly use CPNs with little or no effort at all.

### How to use CPN to model the Immune system function: a novel methodological approach

To develop the adaptive IS response model, we used Colored Generalized Stochastic Petri Nets, in which, as already described, the most common transition rules are defined as stochastic processes. We used the SNOOPY software [20, 21] for developing a proof-of-concept model of the humoral response to an extracellular bacterium. The approach we present can be reassumed as follows:

- Use places to identify cellular or molecular **types**, or subpopulations **types**
- Use transitions to model **events**, such as cellular and molecular state changes, entity interactions, duplication, differentiation, aging and death.
- Use tokens to represent entity **instances**, like cells and molecules (two different tokes represent two **distinct entities**).
- Use colors to define **specific properties** of cellular and molecular entities. In particular, we defined two distinct colorset types (one for cellular entities and one for molecular entities) that include the following colours: STATE, POSITION, LIFETIME and RECEPTOR.

The STATE colorset is used to define internal cell states that determine cells behavior and it is actively used only for cellular types. In the present implementation its possible values are: “resting; active; presenting; memory; plasma”. The LIFE colorset (0…10) is used to define life-time of cellular and molecular entities entities. The RECEPTOR colorset can be used for many functions and its meaning depends on the cellular/molecular entity and transition rule we are dealing with. It can represent:

1. the cellular receptor that is used for the recognition of specific targets by B or T cells;
2. the presented peptide sequence that is presented by Antigen Presenting Cells (APC) like MP;
3. the epitope sequence presented bacteria or antigens in general;
4. the IgG antibody specific binding structure.

The POSITION colorset can be used to model position of cellular entities inside the space or to represent different compartments, i.e. viral infection place and lymph node.

By using this approach and Colored Petri Nets it will be possible to describe in a very general way, with just one transition, the behavior of all the entities that behave similarly (according to some relevant subsets of colors), forgetting about the properties (colors) that are not relevant for the behavior. In other words it will be possible to say, for example, with just only one transition: “All the naive B cells that encounter **in the same position** (check that the pathogen and the B have the same position) and recognize with **sufficient affinity** (check that the B receptor binds with the presented peptide sequence) a specific pathogen will be activated, no matter what is the valule of their real exact position, their value of their real exact receptor or peptide sequence, and how much remaining life they have”. With this approach we will gain a more grained description that introduces, for example, cell receptors and allows to follow entities individually, but at the same time we will obtain a very easy but yet powerful and flexible way to define the general rules that drive the dynamics of the model.

Of course, the definition of the used colors can be adapted to the problem we are dealing with i.e., the POSITION colorset can be removed if we are not interested in modeling space and/or different compartments. On the contrary, we can consider two or tree different position colors (say “X,Y,Z”) to describe two or three dimensional environments.

In our proof-of-concept model we have taken into account 7 different places that are used to represent bacteria, macrophages (MP), T helper cells (TH), B cells (B), Plasma Cells (PB), immunoglobulins class M (IgM) and Immunoglobulins class G (IgG).

As already stated, we defined two distinct product colorsets (multisets) types for all tokens, one type that is mainly used for cellular entities and one type that is used for molecular entities. This first product colorset is composed by four colors: STATE, POSITION, LIFETIME and RECEPTOR, and it is used by TH, B, PB, MP; the second one is composed by three colors: POSITION, LIFETIME, and RECEPTOR and it is used for IgM, IgG, and bacteria. We note here that, even if bacteria are cells and not molecules, we used the second colorset definition for such entities since we were not interested in describing bacteria internal states.

It is worth nothing that in the present model implementation all cells and molecules have distinct product colorsets (i.e. one colorset for TH cells, another one for B cells etc.) in order to improve readability. However, it must be noted all the cellular entities have the same product colorset structure, and this also holds among molecular types. Therefore, it would be possible to define an unique product colorset for all cells and another one for all molecules, since the distinction among different cell types (i.e. B or T cells) or different molecular entities (i.e. IgM and IgG) depends on the context. In fact, since places represent entity types, two identical tokens (i.e., with the same color set and binding) may represent two different kinds of cells if they hold in different places. The transition rules that are based on well-known biological rules define the correct state changes of the system and do not allow the erroneous migration of tokens towards places that represent different entity types.

It will be assumed that for any arc expression we can either have a variable with of a given product colorset type that will bind to any token with the same colorset, or a more complex expression, always based on the colorset definition that will select only tokens that will satisfy some given particular requirements.

For example the arc expression *2’(“active”,p,l,4)*, with *p* representing a variable whose type is POSITION and *l* a variable whose type is LIFE, when placed on an input arc (before a transition) will only accept two tokens with subset colors STATE=“active” and RECEPTOR=“4”. *p* and *l* will bind to any POSITION and LIFE colors of the incoming tokens. In other words, any token representing a cell in the *active* STATE and RECEPTOR *4* will be accepted by the arc expression, no matter what LIFE and POSITION it will have.

The same arc expression when defined on an output arc (after a transition) will produce two tokens with colors STATE=“active”, RECEPTOR=“4”, and POSITION and LIFE colors defined according to the *p* and *l* values. If *p* and *l* binding already occurred, for example on the input arcs of the transition, their value will be already defined and assigned on the tokens that will be produced; if no binding of *p* and *l* occurred on the input arc expressions, their value will be chosen at random inside the set of the allowed POSITION and LIFE colorset values. This example takes into account tokens which represent cellular entities, however, if we remove the STATE color set from the product color set definition, it is also valid for molecular entities.

As already stated, the evolution of the system is driven by transition rules that are defined upon well-known biological rules. Generally, transitions can be used to delineate all the biological scenarios that are needed to describe the evolution of the system. Among such scenarios we recall the appearing of new entities and death of old entities, cellular and molecular behavior changes that include changes in the internal cell state, aging (change in the life counter), processing and presentation of antigenic sequences and movement. Such changes can be the result of transitions that involve one or more entities. Of course it is not possible to describe in detail all the possible biological scenarios that may arise, so we sketch out more explicitly the methodology by showing the most relevant examples.

### Introduction and death of new entities

**Fig. 1:**
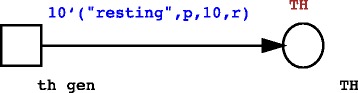
Example of introduction of new entities into the system. 10 new TH cells are introduced at random times into the system as resting cells (STATE = “resting”), with a random position (POSITION = p, where p is defined as a variable of type POSITION), LIFE = 10, and a random receptor (RECEPTOR = r, where r is defined as a variable of type RECEPTOR)

The introduction and of new entities can be modeled as presented in Fig. 1. Usually, new entities can be introduced with stochastic transitions with an initial pre-defined color. For example, as shown in Fig. 1, 10 TH cells are introduced as “resting” cells, with an initial life-time set to 10. Both the RECEPTOR and the POSITION colors are not assigned (POSITION = p and RECEPTOR = r, where p is defined as a variable of type POSITION and r of type RECEPTOR), and this translates into the generation of new cells with random position and receptor, but with a predefined life counter and initial internal state. As it also possible to note from Fig. 1, such kind of transitions does not need any incoming token from a given place to be enabled.

For some scenarios it is requested to model the introduction of new entities at a given time, for example to reproduce the injection of a given treatment or compound. If such situation holds, it is possible to substitute the simple (stochastic) transition with a scheduled transition that allows the definition of an initial time, period, and final time of activation of the transition.

**Fig. 2:**
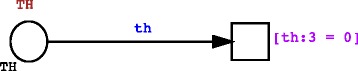
Example of the use of a stochastic transition to model the dead of entities that have completed their life cycle. TH cells that have completed their life cycle will be removed from the system. The transition is enabled if and only if the *guard* condition on the LIFE subset color holds, thus only if the binding on the TH variable (of type TH) is done on a token whose third component element (LIFE) of the product colorset TH is equal to zero (th:3 = 0)

Death of entities will occur for all cells and/or molecules that have ended their life cycle (LIFE = 0). This can be achieved by using transitions with transition guards on the value of the component element that refers to the LIFE counter, as presented in the example shown in Fig. 2. Here, any token can be potentially selected, in fact any token coming from the TH place can bind to the variable th, but the transition will fire only if the guard condition [th:3 = 0] on the transition is satisfied, that is if the third component of TH, representing the LIFE subset color, is 0. If such holds, the input token will be consumed by the transition, and no output tokens will be produced (we have no output arcs).

### Internal state changes

With the preposition “internal state changes”, we define all the events that modify the binding of the components of the product colorsets we have previously defined (i.e., changing in the value of the STATE or LIFE colorsets). Such changes may hold from an immunological point of view for multiple reasons.

**Fig. 3:**
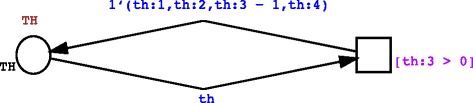
Example of the use of a stochastic transition to model entities aging. The guard on the stochastic transition randomly selects only tokens that represent TH cells whose life is greater than 0 (th:3 > 0), and gives back to the starting place a new token that is identical to the previously selected one, except for the fact that LIFE has been decremented by 1 (1’(th:1,th:2,th:3–1,th:4))

A first example of this scenario can be represented by entities aging. In this case the, LIFE component of entities needs to be decreased to reflect aging. The approach we followed uses stochastic transitions and it is showed in Fig. 3, where we represent a case that shows how TH cells aging can be characterized. Here allow the binding of the variable th with any token with a product colorset TH, we check that its life counter is greater than 0 with the guard expression [th:3 > 0] and, if such holds, we consume the input token and produce, by using the arc expression *1’(th:1,th:2,th:3–1,th:4)*, a new token that has the same STATE (*th:1)*, the same POSITION *(th:2)*, the same RECEPTOR *(th:4)* of the input token, but with a LIFE counter decreased by one *(th:3–1)*.

**Fig. 4:**
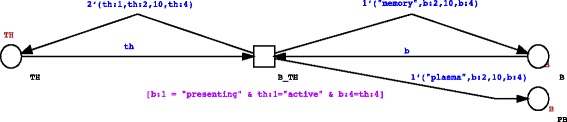
Example of the use of a stochastic transition to model interaction among entities. The guard on the stochastic transition randomly selects only tokens that represent TH cells that are in the active state (th:1=“active”) and B cells that are in the presenting state (b:1=“presenting”). Moreover the activation of the transition is achieved only if both the cells have the same receptor specificity (b:4 = th:4). As a result of the activation of the transition we will obtain duplication of TH cells by giving back 2 identical TH cells to the TH place (2’th), a memory B cell with the same position and specificity, but with renewed life (1’(“memory”,b:2,10,b:4))) and, as a result of a asymetrical division, a new plasma cell (PB) with same receptor and characteristics of the progenitor B cells (1’(“plasma”,b:2,b:3,b:4))

Interaction among two or three different entities represents another example of entity internal state changes. As a result of the interaction of two or more other entities, cells may change their internal state and behavior, molecules like antigens may disappear from the system, and new entities can be introduced. An example is showed in Fig. 4, where we give a possible representation of the interaction between primed TH cells and presenting B cells. Both cells must be in the right internal state to interact; moreover receptor matching is needed to make the interactions possible. As a result of a successful interaction (i.e., availability of tokens in the selected places that satisfy all the requirements in the transition guard) we will obtain duplication of TH cells, and asymmetrical division/differentiation of B cells that will produce a memory B cell and/or a new plasma B cell.

In particular, any token coming from the TH and B places will be a potential candidate to be selected for the binding of the th and b variables, respectively. However, the transition will be enabled only if the guard condition [b:1 = “presenting” & th:1 = “active” & b:4 = th:4] that imposes that: 1) the token representing the B cell is in the “presenting” STATE; 2) the token representing the T helper cell is in the “active” STATE and, 3) there is matching within the involved receptors (b:4 = th:4) is satisfied. As result of the firing of the transition: 1) the input tokens will be consumed; 2) two newborn T helper cells with the same STATE, POSITION and RECEPTOR of the input T helper cell, but with an increased LIFE will be produced (2’(th:1,th:2,10,th:4)) into the TH place; 3) A newborn plasma B cell token with the same RECEPTOR and POSITION of its progenitor, but with LIFE increased to the maximum value will be introduced into the PB (plasma B) place (1’(“plasma”,b:2,10,b:4)); 4) A newborn memory B cell token with the same POSITION and RECEPTOR of the input B cell, but with LIFE increased to the maximum value will be produced into the B place (1’(“plasma”,b:2,10,b:4)).

One of the most important features of the adaptive immune system response is given by the ability to recognize specific targets and characteristics of potential pathogens. This goal is achieved by IS cells using cell receptors that are able to recognize and/or capture specific amino acidic sequences that are present on the surface of infected cells and bacteria.

Such a recognition process can be introduced directly inside transition guards and its definition depends on the RECEPTOR color definition itself. In our example, since we only used a limited set of integer numbers to represent receptors, we supposed that a successful recognition could be achieved only if entities have the same integer number inside the RECEPTOR color. Of course, more complex models of both receptors and receptor recognition functions, like those based on binary strings and hamming distance implemented in the ABM models presented in [2, 3, 6], can be achieved by using specific function definitions based on look-up tables and more complex guards.

**Fig. 5:**
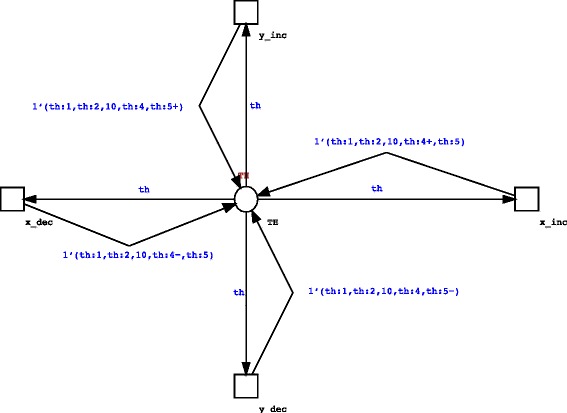
Example of the use of a stochastic transitions to represent 2D random walk over a square grid with toroidal geometry. In this example we suppose that the place that represents TH entities contains tokens defined according the following product colorset: (STATE,LIFE,RECEPTOR, X, Y), with X and Y defined as integer colorsets. Random 2D Movement is achieved by using stochastic transitions. The x_inc and x_dec transitions are used to model movement under the x direction, whereas The y_inc and y_dec represent the movement on the y direction. The predecessor (−) and successor (+) colorset functions are here used to decrement or increment the position indices

Entity position and movement represents another aspect that can be taken into account for modeling IS. In some cases the presence of different compartments or an accurate spatial description can be useful to represent, for example, migration of IS cells towards different compartments (i.e. lymph nodes), tumor growth, and the spread of viral infection. Spatial movement can be achieved as described earlier for entity interactions by using stochastic transitions and guards. In Fig. 5 we show an example on how to implement random walk on two dimensions over a square lattice by using two colorsets X and Y for position. Of course more complex spatial descriptions, like the one presented in [26] for describing reaction diffusion systems, can be used.

We note here that in our proof-of-concept model, even if we predisposed the presence of a POSITION colorset, we did not use it since we did not include any spatial description. It must be highlighted that if space is included inside the model, all transitions guards that are used to model interactions must be revised accordingly to include such an aspect (i.e. to also check that the interacting cells/molecules hold in the same position/compartment).

Delayed events may represent another event that may be critical to represent. For some scenarios it would be of major importance to represent events that require some time to complete, such as antigen internalization and processing by antigen presenting cells like macrophages. Such events can be represented making use of delayed transitions that allow the definition of one or more delay times for the activation of the considered transition.

### Biology implemented into the model

In order to demonstrate the feasibility of the modeling approach, we developed a model of the adaptive immune response whose goal is to reproduce two important IS features: specificity and memory. These two well-known features allow the immune system to recognize and attack specifically a given pathogen thanks to the use o specific cellular receptors (specificity), and to have an enhanced secondary response against a previously encountered pathogen (memory).

**Fig. 6:**
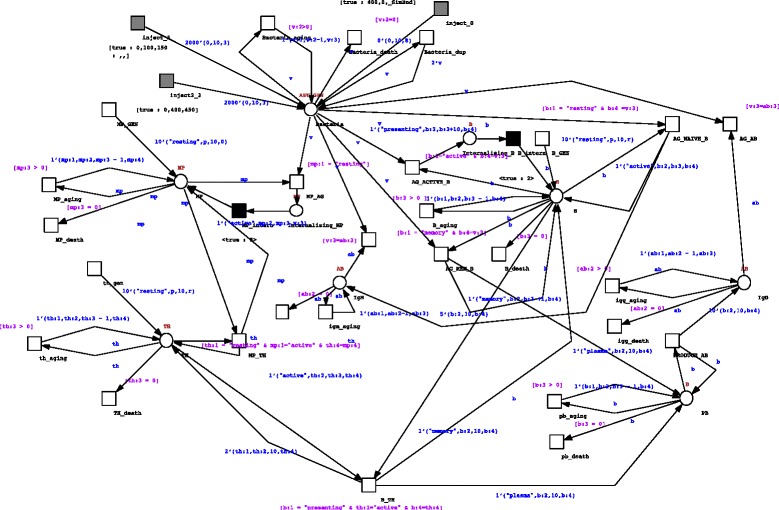
Model of the humoral immune system response. *White circles* represent Places (cellular or molecular families); *white boxes* represent stochastic transitions; grayed boxes that represent scheduled transitions are used to introduce bacteria population at given times; the *two black boxes* that represent delayed transitions are used here to mimic the time needed by macrophages (MP) and B cells to internalize, process and expose antigenic peptides

In this paper we focused on the humoral immune response, that is driven by B lymphocytes and whose final outcome is represented by the production of antibodies. We used SNOOPY [20, 21] to develop the stochastic colored PN according to the approach described earlier. The model is shown in Fig. 6 and is provided online (see Availability of Data and Materials).

The biology implemented into the model can be reassumed as follows. Let us have a given pathogen inside the host i.e., an extracellular bacteria, that is able to replicate. Of course bacteria, like the other cellular entities, are exposed to aging and death. In order to test the presence of the memory and specificity features of the IS response, we supposed two subsequent infection spreads of two different bacterial papulations (with different receptors) into the system at given times using scheduled transitions. Bacteria can be recognized a-specifically by antigen presenting cells like macrophages (MP) through the use of toll-like receptors or also specifically by resting B cells. As a result of such interactions MP that will phagocyte bacteria will be activated and will proceed to process and then expose bacterial peptides complexed with major histocompatibility complex class II peptides. On the other hand, resting B cells that are able to specifically recognize bacterial determinants will be activated and release low affinity antibodies (IgM). Such antibodies will bind with low affinity to bacterial surfaces. However, such a response may be not sufficient to eradicate bacteria. To this end other mechanisms of the IS response are involved. Activated MP will present exposed peptides to specific resting T helper cells (TH) that will be primed; primed B cells that will encounter again bacterial antigens will be further stimulated and will become ready to present bacterial antigens to primed TH cells. When such interaction holds, TH will be duplicate, and B cells will asymmetrically differentiate into plasma B cells (PB) and/or memory B cells. PB will produce specific antibodies (IgG) that will bind with higher affinity to bacterial surfaces, having as a result the death of bacteria (through, for example, activation of complement mechanism). Memory B cells will indeed live longer and will be ready to directly differentiate into plasma cells if future encounters with the same bacteria happen. All interactions can be examined in depth by analyzing the SNOOPY PN model we provided as supplementary material (see Availability of Data and Materials).

## Results and discussion

Immune responses are specific for distinct antigens that are specifically recognized by individual lymphocytes. The parts that the competent immune cells recognize are called determinants or epitopes. This fine specificity exists because individual lymphocytes express membrane receptors that are able to distinguish elusive differences in structure between different epitopes.

The repertoire represents the total number of antigenic specificities of the lymphocytes in an individual. It is extremely large and it has been estimated that the immune system of an individual can discriminate between 10^7^ and 10^9^ distinct antigenic determinants.

**Fig. 7:**
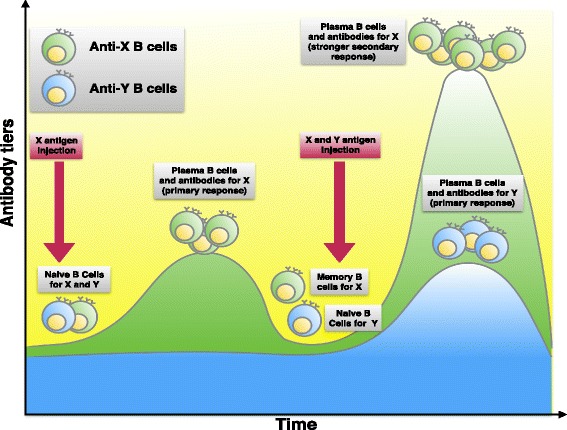
Typical immune system primary and secondary response. Different antigens (X and Y) induce the stimulation of B cells with different specificity, and thus entitle the production of different antibodies (a reflection of specificity). The secondary response to the antigen X is more rapid and stronger than the primary response (underlying memory importance) and is different from the primary response to the antigen Y (again reflecting specificity). Antibody levels decline with time after each immunization

Exposure of the immune system to a foreign antigen enhances its capacity to respond again to that antigen. Responses to second and subsequent exposures to the same antigen, called secondary immune responses, are usually more rapid, larger, and often qualitatively different from the first, or primary, immune response to that antigen (see Fig. 7). Immunologic memory occurs because each exposure to an antigen generates long-lived memory cells specific for the antigen, which are more numerous than the naive cells specific for the antigen that exist before antigen exposure. In addition, these memory cells have special characteristics that make them more efficient at responding to and eliminating the antigen than are naive lymphocytes that have not previously been exposed to the antigen. Figure 7 sketches memory B lymphocytes that produce antibodies that bind antigens with higher affinities than do antibodies produced in primary immune responses. In such an example antigen X is inoculated at the beginning. Immune system competent cells recognize the epitopes of the antigen X and mount the primary immune response, producing specific antibodies against antigen X. Moreover, memory B cells are also produced. Later, another administration is performed. This time, two different antigens are administered, antigen X and Y. The presence of immune memory is highlighted by a larger response. The antigen X is rapidly cleared while the response to the antigen Y is slower, as this is the first time that immune competent cells face with its epitopes.

These features of adaptive immunity are necessary if the immune system is to perform its normal function of host defense. Specificity and memory enable the immune system to mount heightened responses to persistent or recurring exposure to the same antigen and thus to combat infections that are prolonged or occur repeatedly.

Our goal is to demonstrate how the proposed modeling approach based on CPNs can be useful for modeling the IS response. To this end, we developed a proof-of-concept model that is able to qualitatively reproduce the typical primary and secondary IS response that is well known in the literature [1], highlighting the presence of memory and specificity.

While the typical behavior of the immune system response described above is well known and represents one of the hallmarks of immunology, to our best knowledge there exists no specifically validated gold-standard clinical dataset that can be used to quantitatively compare, in a numerical sense and in a precise way, the outcomes of an in vivo/in silico experiment about the general immune system response to a given pathogen. This is due to the fact that the immune system responses are influenced by multiple factors (i.e. the kind of bacterial or viral infection) that can entitle very high fluctuations even among different individuals of the same experimental group. As a result of this, mathematical and computational models are usually tailored to the specific experiment they want to reproduce, and experimental data is usually used to fine-tune the model behavior. In this case, since our goal is to demonstrate how high-level PNs can be successfully applied to qualitatively reproduce specific immune system features rather than numerically reproduce a given experimental setup, we will show how memory and specificity features are coherently reproduced by qualitatively comparing PN based simulation results with those obtained using the UISS simulator, an Agent Based simulation framework that has been developed for years and specialized to model and predict the IS response to different pathologies such as mammary carcinoma [27], lung mestastases [28], melanoma [29], atherosclerosis [30], and to predict and to optimize candidate treatments and novel adjuvants [31].

The time step of all the simulations is Δt = 8 h, as in the ABM models presented in [2, 3, 6]. The total time of all simulations is 600 (approx. 28 weeks). We recall here that the typical adaptive immune system response requires from 2 to 4 weeks.

We used the scheduled transitions to simulate the appearing of bacteria at different times. Since the initial marking of the PN has no tokens, before introducing any bacteria token, we waited a minimal number of time-steps (100 time-steps) to give time to let the PN reach an equilibrium “homeostatic” level for the MP, TH and B places (i.e., a minimal, stable, number of tokes in these places). All the experiments we have done have been repeated 100 times and mean values have been taken into account.

**Fig. 8:**
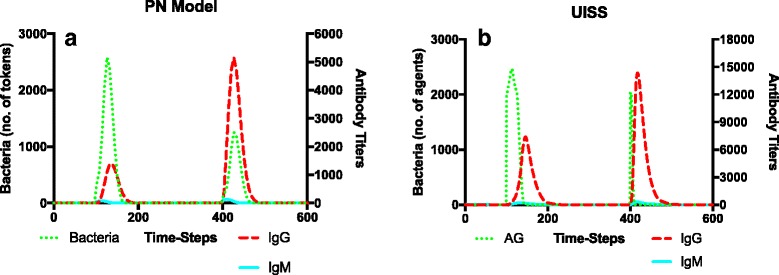
Detailed dynamics of specific immune system entities in the PN model (**a**) and the UISS framework (**b**). At the time of the second bacteria injection (timestep 400), the total level of IgG (*dashed red line*) is much higher in respect to the level measured at the time of the first injection. As a result of this, bacteria (*dotted green line*) are cleared more rapidly at the time of the second injection. Both IgM (*solid cyan line*) and IgG (*dashed red line*) levels obtained with the PN model (**a**) are in good agreement (from a qualitative point of view) with the results obtained by using the UISS framework (**b**)

**Fig. 9:**
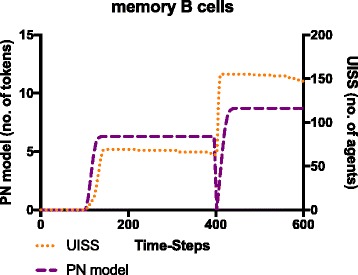
Detailed dynamics of specific memory B cells in the PN model and in the UISS computational framework. Memory B cells levels obtained with the PN model are in good agreement (from a qualitative point of view) with the results obtained by using the UISS framework. The drop in the number of memory B cells in the PN model just after the second injection is caused by the fact that, after the second injection of bacteria, all memory cells are temporarily enrolled into the interaction with bacterial antigens, and rapidly move inside the relative transition (AG_MEM_B transition in Fig. 6). This leaves for a short time the relative place without memory cells. As a result of this, since we actually plot only tokens that are in places, for a very short time the relative curve goes down

As a first experiment we challenged the model to test the induction of memory and compared these first results with those obtained with the UISS framework. To this end we simulated the appearing of a given bacteria (2000 tokens with the binding (0,10,3)) at two different time-steps. We recall here that we did not include the STATE colorset in the bacteria definition. The first appearing of the bacteria (at time-step 100) is done to induce a primary response, whereas the second one (at time-step 400) is done to test the presence of a stronger secondary response. As similar setup (i.e. injection of 2000 bacterial cells at the same time-steps) has been used for the UISS framework also. Results are shown in Figs. 8 and 9. Looking at these figures, it is possible to see how the two modeling approaches show, from a qualitative point of view, show a similar behavior in reproducing the immune system response. In particular, it appears clear that at the time of the second bacteria administration, the total quantity of IgG is in both the models (Figs. 8 (a) and (b)) much higher than the quantity measured at the first injection. Another important aspect is represented by the fact that, as a consequence of the stronger immune response and of a higher peak of IgG, the bacteria are killed more rapidly. Of course some aspects like the total number of antibodies needed and the (lower) time required to clear the second bacterial infection are probably better reproduced by the UISS framework that, after years of work, contains a lot more of biological detail (i.e. all the cytotoxic branch, the presence and function of immunocomplexes, the presence and role of cytokines and chemokines etc.) and a spatial description that, at the present time, has not been enabled in the PN model.

**Fig. 10:**
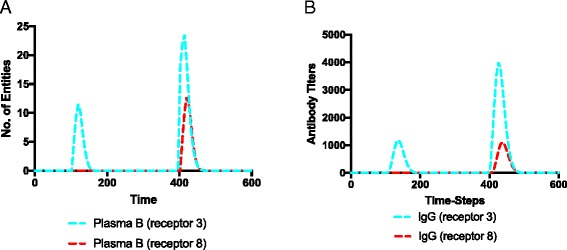
Detailed dynamics of plasma cells and IgG. Both specific plasma B cells (*left panel*, (**a**)) and IgG (*right panel*, (**b**)) are higher for the first bacteria population (with receptor 3, *cyan line*) at the time of the second injection. Furthermore, the response to the second bacteria population (with receptor 8, *red line*) is comparable to the response obtained for the first population (with receptor 3, *cyan line*) at the first time injection. This highlights how the first injection elicits the formation of specific memory B lymphocytes for the first bacteria population only

Once that we have checked that the PN model showed an IS response that is qualitatively in line with the UISS framework, we tested the presence of the specificity feature by injecting two different bacterial populations (identified by a different value in the RECEPTOR subset color, 3 and 8). The first injection of 1000 tokens with the binding (0,10,3) is done at time 100, and then a second injection of both the Bacteria populations (1000 tokens with binding (0,10,3) and 1000 tokens with binding (0,10,8), respectively) is done at time 400. In other words, we reproduced the typical scenario of the immune system response that we have already presented in Fig. 7. In Fig. 10 we show the results obtained with the PN model. As expected, both specific plasma B cells and IgG are higher for the first bacteria population (with binding (0,10,3)) at the time of the second injection. Furthermore, the response to the second bacteria population (with binding (0,10,8)) is comparable to the first population at the first time injection. This highlights how the first injection elicits the formation of memory B lymphocytes that are specific for the first bacteria population only, and thus elicit a stronger immune response for that specific bacteria only.

## Conclusions

Traditional “low-level” PN represent a strong modelling technique for the simulation of networks, concurrent systems and distributed systems in general. Even if PN have been initially used by computer science and engineering theoreticians only, thanks to the introduction of many extensions of the PN framework (which led to the born of “high-level” Petri Nets), PN have also been successfully applied in many other fields, e.g., for the modeling of biochemical pathways. Their initial application to the modeling of the immune system response has been taken into account. However, such an approach did not make inroads, probably because it has not brought any real additional advantage over traditional top-down approaches. For example, the lack of granularity in the description of the biological phenomena may have represented an important missing feature.

Colored PN are a PN extension which may allow to overcome this limit. To demonstrate this, we proposed a methodology to realize detailed models of the immune system response based on (generalized stochastic) CPN, and, to validate our methodology, we presented a model of the humoral adaptive IS response against an extracellular bacteria that is able to reproduce well-known important IS features such as memory and specificity. The proposed methodology has some advantages over classical methodologies. It allows to come close to the granularity allowed by agent based models, by letting the modeler to describe in depth, for example, internal cell states, receptors and complex interactions. At the same time CPN benefit of many types of formal analysis that may permit to analyze some fundamental properties of the biological network under study such as reachability, Liveness, Boundedness, Place invariants, Circuits, T-Invariants and so on [8], making them more similar to differential equations models in this sense.

Furthermore, CPN represent a graphical modeling technique that does not require a strong knowledge of mathematics, like in differential equation based models, or good programming skills, like for ABM models. Thus, CPN based models are easy to design, pre-existing software like SNOOPY or CPN Tools can be used, and life-scientists that already demonstrated able to use PN tools in general for the developing and analysis of many biochemical signaling pathway models may use CPN with little or no effort at all.

Indeed, an additional benefit of the use of CPN is represented by the possibility to realize multi scale models, but with the advantage of using the same modeling technique and software to combine, using hierarchical Petri Nets, intra cellular models (based on signal transduction pathways) and system models (based on the presented approach).
